# Investigating the effect of education based on PRECEDE-PROCEED model on the preventive behaviors of musculoskeletal disorders in a group of nurses

**DOI:** 10.3389/fpubh.2024.1371684

**Published:** 2024-03-18

**Authors:** Tayebeh Rakhshani, Zeinab Limouchi, Hadi Daneshmandi, Amirhossein Kamyab, Ali Khani Jeihooni

**Affiliations:** ^1^Shiraz University of Medical Sciences, Shiraz, Iran; ^2^Fasa University of Medical Sciences, Fasa, Iran

**Keywords:** musculoskeletal disorders, educational intervention, PRECEDE-PROCEED model, nurses, health education

## Abstract

**Background:**

One of the most important occupational complications that could occur in nurses is musculoskeletal disorders. In this study, we designed an educational intervention based on the PRECEDE-PROCEED model to investigate its effects on a group of nurses on preventive behaviors of musculoskeletal disorders.

**Methods:**

A total of 120 nurses working in Izeh City, Iran, participated in this semi-experimental study. The sampling was performed through a convenient sampling method, and the participants were randomly assigned to the experimental and control groups (60 participants for each group). Both groups filled out a questionnaire based on the PRECEDE-PROCEED model before and 2 months after the educational program as part of the data collection process. The data were examined using a paired *t*-test, an independent *t*-test, and a chi-square test after being entered into SPSS version 24.

**Results:**

According to the findings, prior to the intervention, there was no difference between the two groups in terms of their knowledge (*p* = 0.221), attitude (*p* = 0.136), enabling factors (*p* = 0.325), reinforcing factors (*p* = 0.548), self-efficacy (0.421), and behavior (0.257) levels. However, following the intervention, a substantial rise was witnessed in the experimental group in each of the mentioned variables (*p* = 0.001).

**Conclusion:**

In the current study, education based on the PRECEDE-PROCEED model led to the improvement of knowledge, attitude, enabling and reinforcing factors, self-efficacy, and finally preventive behaviors with musculoskeletal disorders in the participants. Considering the importance of the role of health education in promoting behaviors related to musculoskeletal disorders in nurses and the importance of observing related behaviors in preventing long-term complications, the necessity of education in a wider dimension and with different tools is felt more and more in society. Therefore, longer interventions with this aim could be carried out on nurses and other healthcare personnel.

## Introduction

Musculoskeletal disorders refer to any tissue damage to the musculoskeletal system that impairs the functioning of organs (1). These disorders could lead to injuries in the components of the musculoskeletal system, such as joints and bones (1). One of the most common musculoskeletal disorders is caused in the workplace, which is considered one of the main problems of health, disability, and absenteeism in industrialized societies, accounting for about a third of treatment costs (2). These disorders cause 70 million Americans to visit medical centers annually (2), which has made them the second most important occupational disorder (3). Forty percent of all documented occurrences of musculoskeletal problems could lead to permanent disability, and these conditions account for 50% of long-term absences from work (4).

In a study by Jamil et al., 93.2% of the participants had a musculoskeletal disorder, and only a few (32.9%) were aware of workplace ergonomics (5). According to the findings of another study on Chinese computer users, their backs (29.2%), shoulders (10.5%), and hands and wrists (6.2%) were the most commonly damaged areas after a year of work (6). A systematic review and meta-analysis of work-related musculoskeletal disorders in different cities in Iran showed a high prevalence of these issues among employees (7). One of the groups that are exposed to musculoskeletal disorders due to their job responsibilities is nurses. Nurses working in hospitals are considered the main part of the health and treatment system in Iran, and their health status is important in terms of providing healthcare services.

In recent years, due to the COVID-19 pandemic, nurses had to cover long-term shifts, and the lengthened working hours and their increased workload led to serious complications such as stress (8), depression (9, 10), job burnout (11, 12), lack of emotional control (13, 14), reduced resilience (15), and the occurrence of musculoskeletal disorders (16, 17). Most recent studies have focused on nurses and reducing the aforementioned complications. The issue of musculoskeletal disorders in this group has long been the concern of a large group of researchers. The use of educational interventions in order to improve these disorders in nurses has been proposed by the researchers, as these interventions would increase the knowledge, motivation, and ability of nurses to access, evaluate, and use health information (18). In addition, training in managing work and preventing the occurrence of musculoskeletal disorders as one of the life skills increases the strength and abilities of nurses in their jobs (19).

Some previous studies conducted in Iran have shown that education based on health education models has been able to reduce musculoskeletal disorders in nurses and be effective in modulating their pain (20, 21). Among the many models and patterns, the PRECEDE-PROCEED model is an effective one for this purpose (22). In this model, which is also effective in the training of employees, the enablers and reinforcers of behaviors are determined (22). According to this model, the effective factors affecting the results could be determined before designing any intervention. Hence, the PRECEDE-PROCEED model is one of the planning models in health education and health promotion, focusing on changing behaviors, and it has been successful in previous evaluations (23).

The need for an educational program based on an educational model is felt to properly guide nurses in order to prevent the occurrence of musculoskeletal disorders in their workplace. Taking a look at the models of health education, the PRECEDE-PROCEED model is an effective one. Therefore, the aim of this study was to determine the effect of using the PRECEDE-PROCEED model on promoting the preventive behaviors of musculoskeletal disorders in the nurses of Izeh City Hospital in Iran in 2021.

## Methods

### Study design and sampling

This semi-experimental study was conducted among 120 nurses working in Izeh City Hospital, Iran, in 2021. The criteria for entering the study were nurses working in all departments of Izeh City Hospital, having at least 6 months of work experience, consent to participate in the study, and participation in classes. The exclusion criteria were unwillingness to cooperate with the researchers.

According to the findings of Sezgin et al. (24) and taking into account the confidence level of 0.95%, the sample size was determined by using the average comparison formula in two communities, the test power of 80%, and the dropout rate of 10%, resulting in 60 participants in each group. Sampling was performed through a convenient sampling method. In this way, the researcher went to the hospital, got the list of nurses working in different departments, and then approached the nurses and asked them to participate in the study. Then, using computer-generated random numbering, the participants were divided into the experimental and control groups.

### Data gathering tools

To collect information, a questionnaire containing personal information and a questionnaire based on the PRECEDE-PROCEED model were used. The demographic information questionnaire included, demographic characteristics such as age, sex, education, marital status, and income.

### The PRECEDE-PROCEED model questionnaire

This questionnaire included 10 knowledge questions, 10 attitude questions, 9 self-efficacy questions, 5 enabling factors questions, and 10 behavior questions. We assessed the mentioned variables in the participants, as an initial evaluation and to investigate the effect of education on them. Some examples for the questions are provided in Table 1.

**Table 1:** PRECEDE-PROCEED model questions.

Constructs	Questions
Knowledge	Do you know the signs and symptoms of musculoskeletal disorders or have you heard something about them? Do you know about early detection methods for musculoskeletal disorders? In order to detect musculoskeletal disorders on time, what you should check?
Attitude	Exercise is effective in preventing musculoskeletal disorders and unfavorable body positions. The most important consequence of prolonged sitting is musculoskeletal disorders.
Enabling factors	Educational pamphlet influences the prevention of musculoskeletal disorders. Adequate rest is effective in preventing musculoskeletal disorders.
Reinforcing factors	My wife encourages me to exercise. My family encourages me to exercise. The efforts of other colleagues to observe the correct posture in order to prevent pain caused by musculoskeletal disorders.
Self-efficacy	Musculoskeletal disorders can be prevented with the appropriate design of the workstation. The level of knowledge of the personnel and managers regarding the principles of ergonomics in working with computers can prevent musculoskeletal disorders.
Behavior	I exercise at least 3 days a week for 30 min. At my workplace, I walk for 10 min. After sitting behind the system, I put a backrest on my workplace chair. It was designed to prevent back and neck pain.

The range of scores was 10–20 for knowledge, 10–50 for attitude, 9–45 for self-efficacy, 5–25 for enabling and reinforcing factors, and 15–45 for behavior. A higher score in each component indicated a better condition regarding the prevention of musculoskeletal disorders.

### Validity and reliability of the questionnaire

The questionnaire’s validity was assessed using both quantitative and qualitative methods. One epidemiologist, one physician, and eight health education and promotion experts used the questionnaire for its qualitative validity. The content validity ratio and content validity index were utilized to quantitatively verify the validity of the questionnaire.

The content validity ratio was determined by dividing each question into three categories: “the item is necessary, the item is useful but not necessary, and the item is not necessary.” A total of 12 experts assessed the questionnaire using these three spectrums. Using the Lawshe table index, each item larger than 0.56 for 12 persons and the questions pertaining to that item were deemed necessary and retained for further examination.

To assess reliability, the questionnaire was given to 30 participants who met the study’s eligibility requirements in order to ascertain the internal correlation of the tool’s various components. The results of this analysis were analyzed using SPSS 24, and the Cronbach’s alpha coefficient was established for both the questionnaire’s overall content and the PRECEDE-PROCEED model’s constructs. The Cronbach’s alpha value was 0.82 for the full questionnaire, 0.76 for the attitude, 0.81 for enabling factors, 0.86 for reinforcing factors, 0.82 for self-efficacy, and 0.74 for behavior.

### Protocol

First, a questionnaire was given to participants in order for the pre-intervention evaluation. Then, the intervention, which consisted of six sessions lasting between 50 and 60 min, was taken into consideration, over the course of 2 months (one session per week).

Contents were presented by researchers and expert nurses oriented to the topic of the study. Three different major teaching modalities were utilized in this intervention: lectures, Q&A, and group discussions. Initially, participants became familiar with occupational risk factors for musculoskeletal disorders. Classes were held mainly focusing on useful information about musculoskeletal disorders, how to identify them, strengthening and enabling factors, and the barriers to the incidence of this condition. Participants were informed about the correct forms to perform their job duties, how to improve self-care behavior in themselves, and how to eliminate deficiencies in the workplace. At last, one session was specified to review the previous literature. The data were then completed once again and compared with the pre-intervention result 2 months following the intervention (Table 2).

**Table 2:** Educational intervention program.

Session	Aim	Topic	Educational content	Educational method
First	Improving knowledge and attitude of participants toward musculoskeletal disorders	Familiarity with occupational factors of musculoskeletal disorders	Instructing musculoskeletal disorders and occupational risk factors	Speech and question and answer
Second	Introducing the PRECEDE-PROCEED model Identifying enabling and reinforcing factors	Instructing proper techniques at work	Instructing proper techniques for transferring the patient, carrying and lifting equipment, using medical equipment, and working in uncomfortable positions	Lecture, group discussion, and question and answer
Third	Teaching based on the model Introducing self-efficacy	How to perform job duties in the correct way	Educating proper design and arranging the workspace, and arranging the medical equipment correctly	Educational video and poster
Fourth and fifth	Teaching appropriate behaviors using the model	Explaining self-care behaviors	Providing postural and self-care routines to prevent injuries, eliminate deficiencies, and focus on strengths	Group discussion
Sixth	Review and summary	Reviewing the previous materials, summarizing, and final evaluation	A comprehensive review of the provided material	Lecture, group discussion

### Analyzing data

The Kolmogorov–Smirnov test was used to determine the data’s normality before it was entered into SPSS 24. The data were described using frequency, mean, and standard deviation. The analysis was performed using a paired *t*-test, a chi-square test, and an independent *t*-test (*p* < 0.05).

## Results

The study’s control and intervention groups’ demographic data are displayed in Table 3. According to the results of the paired *t*-test, age (*p* = 0.215) and job experience (*p* = 0.648) of participants did not differ between the experimental and control groups. Moreover, the results of the chi-square test revealed no difference in gender (*p* = 0.068), marital status (*p* = 0.426), education (*p* = 0.578), or history of musculoskeletal problems (*p* = 0.159) between the two groups (Table 3).

**Table 3:** Comparison of the frequency distribution of the primary variables of the participants in the two study groups.

Variable		Experimental group (%)	Control group (%)	*p*-value
Age		35.95 ± 4.09	36.88 ± 2.44	0.215**
Work experience		5.37 ± 3.21	6.12 ± 3.25	0.648**
Sex	Female	43 (71.66%)	45 (75%)	0.068*
	Male	17 (28.33%)	15 (25%)	
Marital status	Married	52 (86.66%)	46 (76.66%)	0.426*
	Single	6 (10%)	9 (15%)	
	Other	2 (3.33%)	5 (8.33%)	
Education	Bachelor’s degree	56 (93.33%)	54 (90%)	0.578*
	Masters or higher	4 (6.66%)	6 (10%)	
History of musculoskeletal disorders	No	55 (91.66%)	58 (96.66%)	0.159*
	Yes	5 (8.33%)	2 (3.33%)	

* Chi-square test.

** Independent *t*-test.

The independent t-test found no difference in knowledge (*p* = 0.221), attitude (*p* = 0.136), enabling factors (*p* = 0.325), reinforcing factors (*p* = 0.548), self-efficacy (*p* = 0.421), or behavior (*p* = 0.257) between the groups prior to the intervention. However, following the intervention, there was a noteworthy distinction between the two groups in terms of the mentioned variables (*p* = 0.001) (Table 4).

**Table 4:** Comparison of the constructs of the PRECEDE-PROCEED model before and after the intervention between the two groups.

Constructs	Group	Before intervention (M ± SD)	After intervention (M ± SD)	*p*-value*
Knowledge	Experimental	14.15 ± 2.41	17.21 ± 2.22	0.001
	Control	14.04 ± 2.27	14.21 ± 2.67	0.708
	*P*-value*	0.221	0.001	
Attitude	Experimental	26.81 ± 6.71	37.51 ± 3.25	0.001
	Control	25.97 ± 5.71	26.45 ± 3.33	0.575
	*P*-value*	0.136	0.001	
Enabling factors	Experimental	14.03 ± 3.25	19.19 ± 4.47	0.001
	Control	13.99 ± 2.25	14.04 ± 3.47	0.926
	*P*-value*	0.325	0.001	
Reinforcing factors	Experimental	17.65 ± 2.61	21.16 ± 2.12	0.001
	Control	17.01 ± 2.18	17.27 ± 2.12	0.509
	*P*-value*	0.548	0.001	
Self-efficacy	Experimental	28.12 ± 4.65	35.25 ± 4.61	0.001
	Control	29.12 ± 3.65	28.46 ± 3.61	0.321
	*P*-value*	0.421	0.001	
Behavior	Experimental	26.12 ± 4.29	35.25 ± 5.61	0.001
	Control	27.17 ± 3.29	28.04 ± 4.61	0.236
	*P*-value*	0.257	0.001	

* Independent *t*-test.

## Discussion

This study was implemented with the aim of investigating the effect of education based on the PRECEDE-PROCEED model on the incidence of musculoskeletal disorders in nurses. Based on the results, the educational program resulted in an increase in knowledge in the experimental group compared to the control group. The PRECEDE-PROCEED model includes efforts that seek to understand variables affecting behavior and related processes in health-related fields. This model reveals health interventions and is considered a valuable tool in the research process. This pattern increases a person’s knowledge of the problem and leads to an increase in knowledge and compliance. This finding is consistent with the results of the studies of Sousa et al. (25), Hosseini et al. (26), and Alam et al. (27).

In our study, education led to an increase in attitude in the experimental group compared to the control group. Probably, the increase in attitude in the experimental group was related to the training given. The PRECEDE-PROCEED model is a cognitive model that evaluates the way people respond to a health-threatening factor and leads to an increase in the individual’s attitude when the status quo becomes available. This finding was consistent with the results of Ziam et al.’s studies, with the aim of determining the possibilities and obstacles related to the prevention of musculoskeletal disorders in the older adult (28). This researcher stated that the existence of attitude as a possibility and its absence are known as obstacles that need to be solved by educational intervention in some cases that are related to the individual himself (28). In addition, the results of Pourhaji et al. (29) were consistent with the results of the present study.

Following the intervention, a substantial increase was experienced in the enabling factors in the experimental group compared to the control group. Training based on the PRECEDE-PROCEED model has led to the evaluation of benefits and costs, and for this reason, the trained nurse can reduce costs, prevent complications, and ultimately lead to increased benefits by following some easy training items and preventive behavior in itself. This finding is consistent with the results of Abdul Halim et al. in reducing skeletal problems in hospital employees (30), the study by Jain et al. aimed at the effect of education on physical activity in reducing musculoskeletal problems (31) and the study by Espin et al. aimed at the effect of education in reducing musculoskeletal problems in eldercare workers (32).

After the intervention, a sharp rise was observed in reinforcing factors in the experimental group compared to the control group. This increase can be attributed to the intervention based on PRECEDE-PROCEED, because one of the structures of this model is the knowledge of strengthening factors, and during the training, nurses gained detailed knowledge of strengthening factors in the preventive direction, which in turn, played a role in raising the reinforcing factors of the participants regarding musculoskeletal disorders. This finding is consistent with the results of Toonders et al. on the reduction of musculoskeletal disorders (33) and the study of Alghtani et al. with the aim of exercise-based interventions to reduce back pain in healthcare workers (34).

According to the findings, the intervention led to an increase in self-efficacy in the experimental group compared to the control group. In the topic of musculoskeletal disorders related to work, some obstacles are related to the work itself and the employer, and others, such as how to sit and stand up correctly, how to move the patient, and how to move the wheelchair, are related to the nurse, which can be led by teaching these things to promote self-efficacy in them. This finding is consistent with the results of van de Wijdeven’s (4) and Larinier et al.’s studies (35). Training based on the PRECEDE-PROCEED model led to self-efficacy evaluation in nurses, and finally, the intervention improved self-efficacy.

Finally, in this study, education based on the PRECEDE-PROCEED model has notably promoted the behavior structure in the experimental group compared to the control group. This finding is consistent with the results of Nashaat et al.’s study aimed at the effect of educational intervention on nurses (36) and Abdelaziz et al.’s study aimed at the effect of educational intervention on reducing pain in the older adult with chronic pain (37).

### Strengths and limitations

This study’s strengths include the use of various teaching methods in the educational sessions, the involvement of researchers and nurses in the implementation of the educational intervention, and the design of the educational intervention based on the issues that the majority of nurses face as a result of their jobs.

The rationale for generalizability was highlighted by the short-term follow-up of the training program’s influence and the study’s conclusions’ lack of generalizability to other places.

## Conclusion

The educational intervention in the current study, based on the PRECEDE-PROCEED paradigm, resulted in improved nurses’ knowledge, attitudes, enabling and reinforcing structures, and behavior. The necessity of education in a wider dimension and with different tools is becoming more and more apparent in society, given the significance of the role that health education plays in encouraging behaviors related to musculoskeletal disorders in nurses and the significance of observing related behaviors in preventing long-term complications.

## Data availability statement

The raw data supporting the conclusions of this article will be made available by the authors, upon reasonable request.

## Ethics statement

The studies involving humans were approved by Shiraz University of Medical Sciences. The studies were conducted in accordance with the local legislation and institutional requirements. The participants provided their written informed consent to participate in this study.

## Author contributions

TR: Conceptualization, Methodology, Project administration, Supervision, Visualization, Writing – original draft. ZL: Data curation, Formal analysis, Investigation, Methodology, Resources, Software, Validation, Visualization, Writing – original draft. HD: Conceptualization, Investigation, Methodology, Project administration, Supervision, Validation, Visualization, Writing – original draft. AKa: Data curation, Formal analysis, Investigation, Methodology, Project administration, Resources, Validation, Visualization, Writing – review & editing. AKh: Conceptualization, Data curation, Formal analysis, Funding acquisition, Investigation, Methodology, Project administration, Resources, Software, Supervision, Validation, Visualization, Writing – original draft, Writing – review & editing.
